# Exploring Therapeutic Relationships in Pediatric Occupational Therapy: A Meta-Ethnography

**DOI:** 10.1177/00084174231186078

**Published:** 2023-07-04

**Authors:** Sandrine Gagné-Trudel, Pierre-Yves Therriault, Noémi Cantin

**Keywords:** Child health services, Family, Professional-patient relations, Qualitative research, Rehabilitation, Famille, réadaptation, recherche qualitative, relations entre professionnels et patients, services de santé auprès des enfants

## Abstract

**Background.** Developing strong therapeutic relationships with families is a crucial aspect of pediatric occupational therapy. However, building such relationships is complex as they involve multiple directions of interaction. **Purpose.** To provide a thorough interpretation of children's, caregivers’, and occupational therapists’ experience of the therapeutic relationship. **Method.** A meta-ethnography was realized to synthesize qualitative studies. A systematic search was carried out using five databases from 2005 to 2022. The CAPS checklist was used to appraise included studies’ quality. The analysis was completed using a constant comparison of findings. **Findings.** Three themes emerged from the 14 studies synthesized. The first theme illustrates that the therapeutic relationship can have different meanings depending on the perspective of children, caregivers, or occupational therapists. The second theme explores the components impacting the experience of the relationship. These include the power dynamics, the communication, and respect for diversity. Finally, the third theme illustrates how the relationship can empower positive change. **Implications.** Children, caregivers, and occupational therapists each have a perspective that ought to be heard. Occupational therapists should actively ask for children's and caregivers’ perspectives to encourage power sharing and effective communication. By doing so, occupational therapists can strengthen the therapeutic relationship, which, in turn, promotes positive change.

## Résumé

**Description.** L'établissement de relations thérapeutiques positives avec les familles constitue un aspect crucial de l’ergothérapie à l'enfance. Cependant, l’établissement de telles relations est complexe, car celles-ci impliquent des interactions multidirectionnelles. **But.** Interpréter de façon approfondie l’expérience des enfants, des parents et des ergothérapeutes quant à la relation thérapeutique. **Méthodologie.** Une méta-ethnographie a été réalisée pour synthétiser des études qualitatives. Une recherche systématique a été effectuée dans cinq bases de données entre 2005 et 2022. L'outil CASP a été utilisé pour évaluer la qualité des études incluses. L’analyse a été réalisée à l’aide de la comparaison constante des résultats. **Résultats.** Trois thèmes émergent des quatorze études synthétisées. Le premier thème illustre que la relation thérapeutique a un sens différent selon le point de vue des enfants, des parents ou des ergothérapeutes. Le deuxième thème explore les composantes affectant l’expérience de la relation. Il s’agit notamment de la dynamique de pouvoir, de la communication et du respect de la diversité. Enfin, le troisième thème illustre comment la relation peut favoriser un changement positif. **Implications.** Les enfants, les parents et les ergothérapeutes ont tous un point de vue qui mérite d'être entendu. Les ergothérapeutes doivent activement demander le point de vue des enfants et des parents afin d’encourager le partage du pouvoir et une communication efficace. Ce faisant, les ergothérapeutes peuvent renforcer la relation thérapeutique, ce qui, à son tour, favorise un changement positif.

## The Therapeutic Relationship With Families

The *therapeutic relationship* in occupational therapy is defined as an interactive process between the therapist and a client that takes place within the context of occupation (Restall & Egan, 2021; Taylor, 2020). This term has been widely used in occupational therapy literature since the 1980s (Peloquin, 1990), often interchangeably with the therapeutic alliance and client–therapist interactions. Key aspects of the therapeutic relationship include communication, trust, emotional exchange, and collaboration (Horton et al., 2021; Taylor et al., 2009). By implementing standard strategies and practice models, the therapeutic relationship aims to facilitate occupational engagement and positive therapy outcomes (Taylor, 2020). Indeed, surveys of over 100 occupational therapists showed that more than 90% believed that the relationship improved outcomes related to occupational performance and engagement (Cole & McLean, 2003; Taylor et al., 2009).

The way occupational therapists view the therapeutic relationship has evolved over time. In the mid-20th century, clients (referred to as “patients”) were typically relegated to a passive role, while therapists held hierarchical power and made decisions about treatment without considering the patient’s perspective or needs (Peloquin, 1990). However, the introduction of client-centered practice in the 1980s marked a significant shift in how clients were viewed in the therapeutic relationship (Sumsion, 2000). The respect of clients, the recognition of their knowledge and power sharing were the main characteristics of the relationship (Sumsion, 2000). Although the client was not necessarily as an individual, the idea that the child was the client, separate from his or her family, persisted in pediatric occupational therapy. In the late 1980s, family-centered practice was developed to acknowledge the family as the unit with whom therapists work (Hanna & Rodger, 2002). This framework recognizes the importance of both the child's and the family’s needs and priorities, and places the family as the experts on their child's well-being and development (Rosenbaum et al., 1998). Family-centered practice embraces both the child's and family's needs and priorities. Families are recognized as the individuals who possess the most comprehensive knowledge of their child and are therefore the primary facilitators of their child's functioning (Rosenbaum et al., 1998). In family-centered practice, the establishment of supportive and power-balanced relationships with families is an essential component (Hanna & Rodger, 2002). This means that therapists must respect and value the family's perspectives and insights while working collaboratively with them to identify goals and develop strategies that support the child's overall well-being (Rosenbaum et al., 1998).

Today, family-centered practice, recognized as the best practice, continues to shape the understanding of the therapeutic relationship in pediatric occupational therapy (Novak & Honan, 2019). A recent systematic review aimed to describe the components associated with family-centered practice. The results demonstrated that establishing an equitable and collaborative relationship, along with implementing a reciprocal and collegial approach, is fundamental to family-centered practice (McCarthy & Guerin, 2022).

## The Importance of the Therapeutic Relationship in Pediatric Occupational Therapy

The therapeutic relationship between the therapist and family is important to the process and outcome of occupational therapy. To begin with, the relationship plays a crucial role in promoting a positive perception of the therapy process. When the relationship is positive, it creates a sense of value and co-creation of meaningful experiences from the families’ perspective (King et al., 2020). This positive relationship empowers families and makes them feel optimistic and secure during therapy sessions (D'Arrigo et al., 2020; King et al., 2020).

Furthermore, from the perspective of the families, the therapeutic relationship provides a unique social opportunity for them to express their voice and be heard. This experience is rewarding and motivating for families, as it allows them to participate actively in the therapy process (Curtis et al., 2022). The quality of the relationship between the therapist and the family directly impacts the meaningfulness of the therapy process, underscoring the importance of establishing a strong and positive therapeutic relationship (King et al., 2020).

A supportive therapeutic relationship can also impact families’ outcomes. McCarthy and Guerin (2022) conducted a systematic review of 42 studies realized in early intervention settings to document such impacts. According to their results, a supportive and respectful relationship, with family-centered intervention strategies, promotes children's social, functional, and motor outcomes. It also improves caregivers’ well-being, knowledge, and empowerment. In a systematic review conducted by Kruijsen-Terpstra et al. (2014), 13 studies in rehabilitation settings for young children with cerebral palsy were included, and similar findings were reported. Their analysis underscored the significance of building a relationship based on respect, trust, support, and shared decision-making in combination with comprehensive care. This approach was found to improve the health and well-being of the children, as well as the outcomes of their caregivers. Thus, the importance of fostering a positive therapeutic relationship cannot be overstated, as it has implications for multiple aspects of the therapy process.

Despite consensus on the importance of establishing supportive relationships in occupational therapy, it remains complex. Establishing these relationships with families was identified as an important challenge by occupational therapists (Pereira & Seruya, 2021). The challenge lies in managing a multidirectional relationship in which all family members have distinct values, views, needs, and goals. Occupational therapists need to consider family dynamics as they support meaningful participation and cooperation between each member towards shared goals (Hanna & Rodger, 2002; Pereira & Seruya, 2021). Given the complexity of establishing an effective relationship, it sometimes remains an ideal to be achieved instead of a reality. Unfortunately, some family members report feeling dissatisfied with their opportunities to participate in the relationship (Stefánsdóttir & Egilson, 2016).

## The Current State of Research

Due to its complex nature, the therapeutic relationship in occupational therapy has been the focus of several studies in the literature. Qualitative studies, in particular, are valuable for gaining a deep understanding of this relationship’s complexity. However, to date, no meta-synthesis has been conducted to integrate the current qualitative findings on the therapeutic relationship in the context of pediatric occupational therapy practice.

Many qualitative studies have separately explored the experiences of children, caregivers, or occupational therapists. However, since the therapeutic relationship is multidirectional, it is essential to consider the perspectives of all groups involved, including children and caregivers. Therefore, the aim of this study is to provide a thorough interpretation of the experiences of children, caregivers, and occupational therapists regarding the therapeutic relationship in the context of pediatric occupational therapy services. By bringing together the perspectives of all groups, a deeper understanding of the therapeutic relationship can be gained, and how it can be optimized for effective pediatric occupational therapy practice.

## Method

An interpretative synthesis approach, specifically meta-ethnography, was used for synthesizing qualitative research. This meta-ethnography was registered with the International Prospective Register of Systematic Reviews (PROSPERO: CRD42022326870). The Meta-ethnography Reporting Guidance (eMERGe) standards (France et al., 2019) were used for reporting this synthesis. The meta-ethnography produces new interpretations that go beyond individual studies and thus contribute to conceptual and theoretical development in a field (France et al., 2019). The originators of meta-ethnography, Noblit and Hare (1988), described meta-ethnography as a means to “Making a whole into something more than the parts alone imply” (p. 28). It is frequently used for qualitative synthesis in healthcare research (France et al., 2019), and in occupational therapy as well (Zedel & Chen, 2021). This synthesis approach has already been identified as useful and accessible to inform occupational therapy practice (Gewurtz et al., 2008). Moreover, given the complexity of the therapeutic relationship, meta-ethnography is relevant since it allows for new interpretations, especially because a vast number of primary studies on the subject exists.

### Data Collection

**Search strategy.** Data collection began with a search using keywords related to therapeutic relationship, occupational therapy, children, and qualitative inputs. Data search was conducted using five electronic databases (CINAHL, Google Scholar, Medline, PsycINFO, and Scopus) from January 1, 2005 to May 16, 2022. Terms were adapted as necessary for effective searches in all databases. The keywords and bibliographic databases were reviewed by a research librarian. The full-search equation is available in Supplemental Files. Manual searches in the reference lists of included studies and in seven relevant journals (e.g., *Physical and Occupational Therapy in Pediatrics*) were used for completeness.

**Study selection.** Reference management software (Endnote ×9) was used to facilitate the selection process. It began with the removal of duplicates. Then, titles and abstracts were read while screening by the first author. Study selection was completed with a full-text review. Studies were selected for inclusion by the first author, and any uncertainty was discussed with the second author. The eligibility criteria are described in Table 1.

**Table 1:** Study Characteristics Used as Criteria for Eligibility

Type of studies	Studies available in English or French, peer-reviewed, and published after 2005 were eligible for inclusion. The studies needed to be published after 2005 to collect data reflecting contemporary practice. Conference abstracts were excluded from the study.
Participants	Studies must investigate the experience of the therapeutic relationship from the perspective of: Children ( ≤ 18 years old): No criteria about the diagnosis were used. Caregivers of child receiving occupational therapy services: The term “caregiver” refers to the primary carer of the child. Occupational therapists working in pediatric settings: When the perspective of occupational therapists is solicited along with other professionals, they must represent at least 50% of the sample or the participant's profession must be clearly identified in the results. Studies collecting the perspective of occupational therapy students were excluded from the study.
Study design	Qualitative study designs (primary research) were eligible since they helped understand the experience of the therapeutic relationship in occupational therapy. Qualitative research is particularly relevant when trying to understand the complexity of human interactions. Examples of eligible study designs included phenomenology, grounded theory, and qualitative descriptive studies. Case studies and mixed design studies were excluded from the study.
Outcomes	Caregivers’, children's and occupational therapists’ experiences of relationship, or any other terms referring to subjective measures of relationship were eligible.

**Critical appraisal.** A formal assessment of the methodology was completed. The Critical Appraisal Skills Programme (CASP, 2022) checklist was used for quality assessment. The CAPS checklist has already been used for appraising the quality of studies for meta-ethnography (France et al., 2019). It helped in the interpretation of the included studies’ potential contribution to the synthesis. Each included study was evaluated as a key paper (KP) with conceptual richness and rigor in the method; a satisfactory paper (SAT), or a paper that is “fatally flawed” from a methodological standpoint (FF; Malpass et al., 2009). The categorization of each included study is available in Supplemental Files. The first author appraised papers, and any uncertainty was discussed with the second author to make a final decision.

### Data Analysis

The data analysis started with reading the studies. The first author carefully read and re-read the full-text papers to become familiar with the content of the studies. At the same time, the first author started to extract participants’ views and interpretations of their experience of the therapeutic relationship in their words (first-order constructs) and themes developed by the authors of primary studies (second-order constructs). This process was facilitated by NVivo 12 software. The software was used to keep track of second-order constructs and memos. Then, a constant comparison was performed to examine how studies were related. The meanings of the second-order constructs from the perspective of the different parties (e.g., caregivers) were compared by creating a pile/group with NVivo tools. Then, the first author used concept maps to show how second-order constructs were related within each paper, after which a refined set of themes (third-order constructs) was generated. The overarching comprehension of the therapeutic relationship was captured by comparing findings of original studies, third-order constructs, and memos. The process of synthesizing the translations was carried out by the first author and discussed with the third author.

## Findings

### Characteristics of the Included Studies

**Figure 1.**
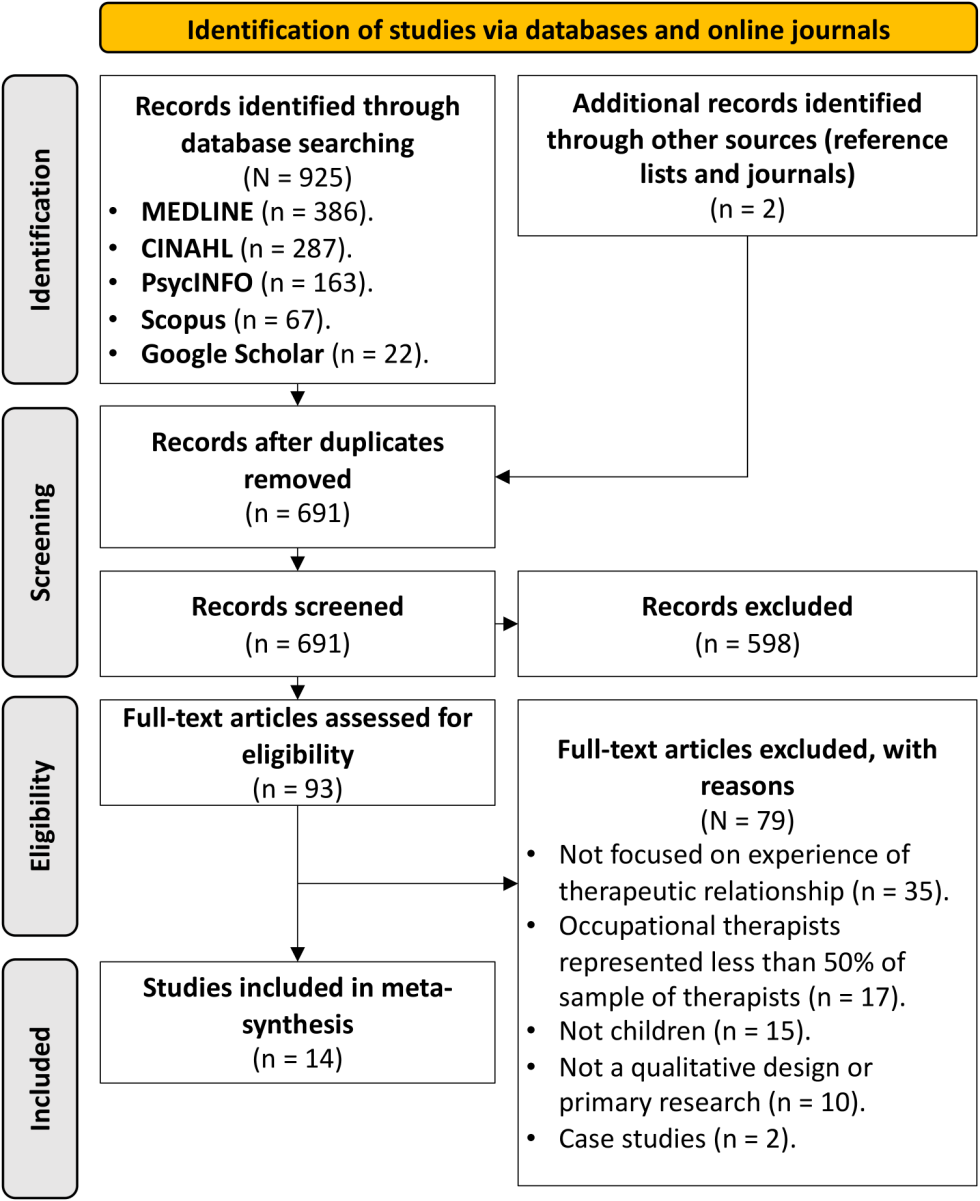
Flow diagram of a systematic review search process.

As shown in Figure 1, the selection process is depicted in a flow diagram. Fourteen articles published between 2007 and 2021 were included in this meta-ethnography.

The research designs of included studies were descriptive qualitative research (*n* = 8), phenomenology (*n* = 4), ethnography (*n* = 1), and grounded theory (*n* = 1). Supplemental Files provide details on included studies (e.g., location, goals, design, main results, etc.). As shown in Supplemental Files, three studies were categorized as KP, eight studies were categorized as SAT, and three studies were categorized as FF. The studies provided perspectives of occupational therapists (*n* = 9), caregivers (*n* = 5), and children (*n* = 3). Their characteristics are presented in Table 2.

**Table 2:** Participants Characteristics in Included Studies

Occupational therapist's characteristics	Caregivers’ characteristics	Children's characteristics
Clinical settings: Public healthcare services and school-based services. Clinical experience: Between 1 and 41 years.	Mainly mothers. Limited sociodemographic data documented.	Age: Between 6 and 18 years old. Diagnoses: Cerebral palsy, autism spectrum disorder, neuromuscular condition, or mental health disorder.

### Main Findings

The aim of this meta-ethnography is to provide a thorough interpretation of the experiences of children, caregivers, and occupational therapists regarding the therapeutic relationship. The study revealed three main themes: (1) “Relationship's meaning is different depending on the perspective,” (2) “Pivotal components impact the experience of the relationship,” and (3) “Therapeutic relationship empowers change.”

**Relationship's Meaning Is Different Depending On the Perspective.** The meaning of the therapeutic relationship is experienced differently depending on the perspective of the children, caregivers, or occupational therapists. The children's perspective about the meaning of the therapeutic relationship is presented, followed by the perspective of caregivers and occupational therapists.

***Children's experiences: be heard.*** From the perspective of children, engaging in a therapeutic relationship means their voice needs to be heard. Children referred to the experience of having their voice heard with respect. They want to be heard through nonjudgmental collaboration while focusing on their unique occupational needs (Shea & Jackson, 2015). The experience of being heard by the occupational therapist was also very significant as it allowed them to express themselves and communicate more effectively (Shea & Jackson, 2015). Being heard is also meaningful given it has a positive impact on children reaching their goals (O’Connor et al., 2021). However, some children reported negative experience where the therapist made decisions for them or told them what to do (Shea & Jackson, 2015). Additionally, some children expressed their desires about the direction of therapy but were not listened to, as noted by O’Connor et al. (2021).

***Caregivers’ experiences: evolve within a climate of trust.*** From the perspective of caregivers, the therapeutic relationship evolves gradually, along with the development of trust (Lindsay et al., 2014; Nelson & Allison, 2007). Also, the relationship has strong emotional value. They believe that a climate of trust acts to foster the therapeutic alliance, meaning that trust strengthens engagement and collaboration with the occupational therapist (Smit et al., 2018). When caregivers feel they can trust their therapist, they become more comfortable expressing themselves about therapy and are empowered to change its course (King et al., 2021). Experiences based on trust are closely linked with empowerment. A climate of trust and emotional safety allows caregivers to take control and act in the relationship (Smit et al., 2018). Trust is built through consistency and communication (Keiter Humbert et al., 2020).

***Occupational therapists’ experiences: balance with professional responsibility.*** From the perspective of occupational therapists, the experience of the therapeutic relationship means “balancing a positive relationship with professional responsibility” (Reeder & Morris, 2018, p. 373). They were concerned with establishing a positive relationship with families, while recognizing their professional obligations. Occupational therapists must maintain their relationships within professional boundaries and combine this responsibility with the needs of the family, as illustrated by the theme “Going beyond and above” (Keiter Humbert et al., 2020, p. 80). In addition, occupational therapists often navigate between their responsibilities to share difficult information (e.g., poor functional prognosis for the child) and to maintain a good relationship with the family. They recognize that providing difficult information can put the relationship, family engagement, and even caregiver’s hope for the future at risk (Keiter Humbert et al., 2020).

**Pivotal Components Impact the Experience of the Therapeutic Relationship.** This meta-ethnography highlights three pivotal components related to the experience of the therapeutic relationship. These components included the negotiation within the power relationship, the mutual and clear communication, and the collaboration with respect for diversity.

***Negotiate within the power relationship.*** The power relationship emerged as a recurring theme when children, caregivers, and occupational therapists shared their experiences of the therapeutic relationship. Families valued holding power in the relationship and wanted occupational therapists to recognize their choices and autonomy (O’Connor et al., 2021; Shea & Jackson, 2015). Some families remained with the feeling that the occupational therapist was the expert and should lead the decision-making (Andrews et al., 2013; King et al., 2021; O’Connor et al., 2021). However, the general experience of families suggests that they wanted to have control and be involved in decisions. They related to experiences in which they wanted to hold more power in the relationship, including being more involved in decision-making, but were kept from doing so (Kruijsen-Terpstra et al., 2016). In these instances, negative experiences occurred when occupational therapists attempted to make choices for them (Shea & Jackson, 2015). Occupational therapists had a common goal of distributing power with families (Tam-Seto & Versnel, 2015). However, they identified barriers to achieving this goal, particularly in situations where decisions posed security risks, when the child had a cognitive limitation or was young (O’Connor et al., 2021; Tam-Seto & Versnel, 2015). Occupational therapists recognized their significant power in the relationship, which manifested in their tendency to guide the family toward specific goals (O’Connor et al., 2021) and their choice not to share certain information entirely (Kruijsen-Terpstra et al., 2016; Tam-Seto & Versnel, 2015). However, they also acknowledged that lack of openness in sharing information was detrimental to establishing equal relationships (Kruijsen-Terpstra et al., 2016).

***Communicate mutually and clearly.*** This meta-ethnography provides a better understanding of the experience of communication as defined by the individuals experiencing it. Communication was a recurring theme as caregivers and occupational therapists shared their perspectives on the therapeutic relationship. Communication was seen as a process between caregivers and occupational therapists. Children’s perspective of their experience of communication in the therapeutic relationship was not collected in the included studies. For caregivers, positive experiences of the therapeutic relationship were associated with a mutual process of communication, rather than the occupational therapist telling them what to do (Kolehmainen et al., 2010; Kruijsen-Terpstra et al., 2016; Nelson & Allison, 2007). The “shared understanding” was considered important from both perspectives (Kolehmainen et al., 2010; Lindsay et al., 2014). Caregivers’ opportunities for communicating were influenced by the beliefs, held by both themselves and the therapists, that the caregivers knows their child best, and the comfortable space to ask questions (Fingerhut et al., 2013; Lindsay et al., 2014). The clarity of the communication was also identified as an important factor in the caregivers’ experience (Kruijsen-Terpstra et al., 2016). The use of jargon during discussions or misunderstandings led to negative emotions such as frustration, anxiety, distress, and feelings of incompetence (Andrews et al., 2013; O’Connor et al., 2021; Smit et al., 2018).

**Collaborate With Respect for Diversity.** Children, caregivers and occupational therapists emphasized the importance of respecting diversity in establishing a therapeutic relationship. This meant nonjudgmental collaboration and unconditional acceptance of their diverse interests, cultures, and personalities (Shea & Jackson, 2015). Occupational therapists also recognized the value of collaboration with families from diverse backgrounds in establishing effective therapeutic relationships. The families’ cultures, including language spoken at home, gender relations, meaning of nonverbal language, and value placed on the child's independence, impacted their possibility to collaborate (Andrews et al., 2013; Lindsay et al., 2014; Nelson & Allison, 2007). This impact was increased when families and occupational therapists were not sharing the same cultural references. In such cases, occupational therapists sought resources to overcome barriers and ensure that they are collaborating in a way that respects diversity and makes families feel comfortable (Fingerhut et al., 2013; Kennedy et al., 2020; Lindsay et al., 2014; Nelson & Allison, 2007). Therapists’ resources included flexibility, creativity, and patience (Nelson & Allison, 2007). Access to external resources such as continuing education, interpreters, material in different languages, and time was also viewed as crucial (Fingerhut et al., 2013; Lindsay et al., 2014). However, they often face challenges in accessing these resources (Lindsay et al., 2014). Finally, respecting the diversity was also important considering the strong cultural dimension of the relational context in occupational therapy (e.g., addressing occupations of eating, playing, or sleeping; Keiter Humbert et al., 2020; Lindsay et al., 2014).

**Therapeutic Relationship Empowers Change.** The therapeutic relationship between children, caregivers, and occupational therapists is a powerful catalyst for change. Nelson and Allison (2007) highlighted the theme that “relationships improve outcomes” (p. 63). The theme explored how the therapeutic relationship itself can have more impact than the “activity” of therapy. Interactions with occupational therapists improved empowerment (Fingerhut et al., 2013) and understanding of the family (Kolehmainen et al., 2010; Smit et al., 2018). Supportive therapeutic relationships were seen as a key factor in accelerating occupational performance outcomes for the child (Smit et al., 2018) and driving meaningful change (King et al., 2021). Indeed, the relationship fosters collaboration and engagement in therapy. The engagement was change-inducing because it influenced progress and real-life outcomes. It leaded to changes in “implementation intentions, planning, beliefs about self-efficacy, and real-world practice” (King et al., 2021, p. 2361). Occupational therapists also experience professional and personal growth through their relationship with families. The results of Keiter Humbert et al. (2020) revealed the theme “learning from one another” (p. 81) in which occupational therapists noted that they gained insights and awareness while collaborating with families. Children, caregivers, and occupational therapists are all experiencing change through the therapeutic relationship.

## Discussion

The aim of this meta-ethnography was to provide a thorough interpretation of children's, caregivers’, and occupational therapists’ experience of the therapeutic relationship. This study enabled the integration of findings from multiple qualitative studies conducted in diverse contexts and using various methods. The findings of the study prompt two main reflections. Firstly, the study highlights the importance of children's voices in the therapeutic relationship. Although children view the therapeutic relationship as a valuable opportunity to express themselves, their voices are not always given due consideration. Secondly, the study reveals the issue of power dynamics in the therapeutic relationship, where occupational therapists hold a significant amount of power. Addressing these power imbalances is crucial to promoting more equitable and collaborative therapeutic relationships.

### Raising Children's Voices in the Therapeutic Relationship

Children associated the therapeutic relationship with a meaningful social experience of having a voice and being heard. However, the opportunity to collect their perspective was often glossed over by occupational therapists. The information sharing was also seen as a process between caregivers and therapists, in which the children were not involved. These findings also resonate with the scoping review of Pritchard-Wiart and Phelan (2018), noting that children's voices are not sufficiently heard in rehabilitation services. This is a considerable concern given the importance of listening to the voices of children in the therapeutic relationship. Curtis et al. (2022) conducted a systematic review that included 19 studies (quantitative, qualitative, and mixed methods studies) exploring children’s voice in occupational and physical therapy. Their results demonstrated how listening to children voices enhanced the child's motivation, as well as their sense of being heard, and valued. The authors further emphasized that hearing children's voices is critically important to keep children engaged in the relationship. Thus, despite the crucial role of hearing children's voices in the therapeutic relationship, occupational therapists seem limited in their ability to do so.

Occupational therapists are encouraged to raise the voice of children in the therapeutic relationship. Occupational therapists should actively search for children's perspectives during therapy (Lundy, 2007). For example, they can use standardized tools to collect children's perspectives regarding their goals, such as the Perceived Efficacy and Goal Setting System (Missiuna et al., 2006) and the Canadian Occupational Performance Measure (Verkerk et al., 2021). Occupational therapists play a crucial role in enabling children to express themselves and ensuring that their voices are heard and taken seriously. However, as highlighted by this meta-ethnography, simply offering children the opportunity to speak is not enough. Some children expressed themselves about their vision but were not listened to. Thus, it is important to ensure that their participation is oriented toward active engagement (Lundy, 2007). The absence of therapeutic actions to pursue their vision, following their consultation, can be more damaging than nonparticipation. The invitation to participate creates expectations that, if subsequently unmet, can lead to frustration in children (Gal, 2017). Thus, it is important to give children the opportunity to express themselves and to make their voice meaningful. In sum, occupational therapists must be mindful not only of providing child-friendly services, but also of creating a culture of respect that empowers children to express themselves meaningfully.

### Moving Toward Collaborative Relationship-Focused Occupational Therapy

This meta-ethnography highlights the growing attention given to power dynamics in therapeutic relationships. Occupational therapists recognized that power imbalance within families remains. They acknowledged their power over the choice of some therapeutic goals, especially when family member had cognitive limitations, when the child was young, or when the goals conflicted with their values, such as safety. It is apparent that the values held by occupational therapists are informed by a variety of factors, including the dominant social views that exist within their field (Restall & Egan, 2021). However, this has the potential to reinforce power imbalances, particularly in situations where families hold a lower social position due to factors such as their age, ability, class, colonial history, and ethnicity (Hammell, 2015). However, their discourse was silent about their acknowledgment of how their social positions affect power dynamics. This silence in the broader occupational therapy literature was also observed by Restall and Egan (2021).

Based on our findings, occupational therapists were diligent in establishing respectful relationships with families from diverse backgrounds. However, establishing respectful relationships is insufficient for genuinely sharing power. Thus, the collaborative relationship-focused practice is a relevant avenue for raising awareness about power issues. By encouraging critical reflection on how occupational therapists relate to families, as well as their knowledge, histories, cultures, and economic structures, this practice can help to address power imbalances within the therapeutic relationship. It encourages occupational therapists to do the hard work of identifying their social position and the families’ position impacting relationship building. Client-centered and family-centered practice remains good starting points for addressing power sharing. However, the reflections generated by collaborative relationship-focused practice appear essential to understand the contexts in which the therapeutic relationship occurs and to address adequately power issues (Restall & Egan, 2021).

### Limitations

Three main limitations impact the quality of this meta-ethnography. Firstly, the included studies mainly explored the perspective of occupational therapists. The children's and caregivers’ perspectives were underrepresented in included studies. Also, the perspective of caregivers and children with many backgrounds were not represented. In future research, it would be relevant to collect their perspective regarding their experience of the relationship. Secondly, three included studies were categorized as FF from a methodological standpoint. However, removing studies rated as FF had no significant impact on the themes, except on the theme “Children's experiences: Be heard.” Rigorous qualitative studies are needed to capture the perspective of children around the therapeutic relationship in occupational therapy. Thirdly, only the first author conducted this meta-ethnography. Accordingly, different perspectives did not inform the review process. As a result, the review might be influenced by the author's background and own experience as an occupational therapist. Writing the memos helped the author question how her pre-existing knowledge may have influenced the review process.

## Conclusions

This meta-ethnography aims to deepen the understanding of the therapeutic relationship from the perspectives of children, caregivers, and occupational therapists. It reveals that these individuals have different interpretations of the therapeutic relationship. Children associate the relationship with an experience of being heard, while caregivers put a strong emphasis on climate of trust. For occupational therapists, the relationship is more about balancing their professional responsibilities with building positive relationships with families. The experience of the therapeutic relationship is significantly impacted by power dynamics, communication, and respect for diversity. Finally, children, caregivers, and occupational therapists emphasize the positive outcomes following their engagement in the therapeutic relationship.

## Key Messages

- The therapeutic relationship in occupational therapy is important, complex, and its impacts are increasingly understood.
- In clinical practice, occupational therapists should listen to children to make their voices meaningful.
- Qualitative research in occupational therapy would benefit from the perspective of families, including children and caregivers from multiple backgrounds.

## Supplemental Material

sj-docx-1-cjo-10.1177_00084174231186078 - Supplemental material for Exploring Therapeutic Relationships in Pediatric Occupational Therapy: A Meta-Ethnography Supplemental material, sj-docx-1-cjo-10.1177_00084174231186078 for Exploring Therapeutic Relationships in Pediatric Occupational Therapy: A Meta-Ethnography by Sandrine Gagné-Trudel, Pierre-Yves Therriault and Noémi Cantin in Canadian Journal of Occupational Therapy

sj-docx-2-cjo-10.1177_00084174231186078 - Supplemental material for Exploring Therapeutic Relationships in Pediatric Occupational Therapy: A Meta-Ethnography Supplemental material, sj-docx-2-cjo-10.1177_00084174231186078 for Exploring Therapeutic Relationships in Pediatric Occupational Therapy: A Meta-Ethnography by Sandrine Gagné-Trudel, Pierre-Yves Therriault and Noémi Cantin in Canadian Journal of Occupational Therapy
