# Supplementary material for: Exploring Therapeutic Relationships in Pediatric Occupational Therapy: A Meta-Ethnography
Source: Can J Occup Ther. 2023 Jul 4;91(1):78–87. doi: 10.1177/00084174231186078 (PMC10903134; doi:10.1177/00084174231186078)
Supplement: sj-docx-1-cjo-10.1177_00084174231186078 - Supplemental material for Exploring Therapeutic Relationships in Pediatric Occupational Therapy: A Meta-Ethnography [file sj-docx-1-cjo-10.1177_00084174231186078.docx]

**Table**

*Search Strategies*

| Electronic databases | 1^st^ keywords group | 2^nd^ keywords group | 3^rd^ keywords group | 4^rd^ keywords group | | Limiters |
| --- | --- | --- | --- | --- | --- | --- |
| CINAHL Complete (from EBSCO) | "**Professional-Client Relations**" OR "**Professional-Family Relations**" OR "**Professional-Patient Relations**" OR "**Family Centered Care**" OR "professional-family relation" OR "family-professional relation" OR "client-professional relation" OR "professional-client relation" OR "parent-therapist interaction" OR "family-professional relation" OR "therapeutic relation" OR "therapeutic relationship" OR "therapeutic relationships" OR alliance OR collaborati* OR "family-centered" OR "family-centred" OR "family centered" OR "family centred" OR fcp OR fcc OR "client-centred" OR "client centred" OR "client-centered" OR "client centered" OR "decision-making" OR "decision making" OR "goal setting" | "**Occupational Therapy**" OR "**Pediatric Occupational Therapy**" OR "occupational therapist" OR "occupational therapists" OR ot OR ergothérap* | **Child** OR **Adolescence** OR **Infant** OR "**Child, Disabled**" OR children OR pediatri* OR paediatri* OR youth* OR toddler* OR adolescen* OR enfant* | "**Qualitative Studies**" OR qualitati* OR interview* OR perception* OR view* OR experience* OR perspective* OR phenomenolog* | | - All child. - Publication date : After 2005. - Language : English and French. - Source types : Academic journals. |
| MEDLINE (from EBSCO) | "**Professional-Patient Relations**" OR "**Professional-Family Relations**"  OR "professional-family relation" OR "family-professional relation" OR "client-professional relation" OR "professional-client relation" OR "parent-therapist interaction" OR "family-professional relation" OR "therapeutic relation" OR "therapeutic relationship" OR "therapeutic relationships" OR alliance OR collaborati* OR "family-centered" OR "family-centred" OR "family centered" OR "family centred" OR fcp OR fcc OR "client-centred" OR "client centred" OR "client-centered" OR "client centered" OR "decision-making" OR "decision making" OR "goal setting" | "**Occupational Therapy**" OR  "occupational therapist" OR "occupational therapists" OR ot OR ergothérap* | **Child** OR **Infant** OR "**Disabled Children**" OR **Adolescent** OR pediatric OR  children OR pediatri* OR paediatric OR youth* OR toddler* OR adolescen* OR infant OR enfant* | | **Qualitative Research** OR qualitati* OR interview* OR perception* OR view* OR experience* OR perspective* OR phenomenolog* | - All child. - Publication date : After 2005. - Language : English and French. - Source Types : Academic Journals. |
| APA PsycINFO (from EBSCO) | "**Therapeutic alliance**" OR "Professional-Family Relation"  OR "professional-family relation" OR "family-professional relation" OR "client-professional relation" OR "professional-client relation" OR "parent-therapist interaction" OR "family-professional relation" OR "therapeutic relation" OR "therapeutic relationship" OR "therapeutic relationships" OR alliance OR collaborati* OR "family-centered" OR "family-centred" OR "family centered" OR "family centred" OR fcp OR fcc OR "client-centred" OR "client centred" OR "client-centered" OR "client centered" OR "decision-making" OR "decision making" OR "goal setting" | "**Occupational Therapy**" OR "occupational therapist" OR "occupational therapists" OR ot OR ergothérap* | **Pediatrics** OR  child* OR pediatric OR paediatric OR youth* OR toddler* OR adolescent* OR infant OR enfant* | |  | - Publication date : After 2005. - Source Types : Academic Journals. - Methodology : Qualitative study. |
| Google Scholar | Therapeutic relationship | Occupational therapy | Child | | Qualitative | Articles de revue.  2005-2022.  Trier par pertinence.  First 22 articles in order of relevance. |
| Scopus | "Professional-Client Relations" OR "Professional-Family Relations" OR "Professional-Patient Relations" OR "Family Centered Care" OR "professional-family relation" OR "family-professional relation" OR "client-professional relation" OR "professional-client relation" OR "parent-therapist interaction" OR "family-professional relation" OR "therapeutic relation" OR "therapeutic relationship" OR "therapeutic relationships" OR alliance OR collaborati* OR "family-centered" OR "family-centred" OR "family centered" OR "family centred" OR fcp OR fcc OR "client-centred" OR "client centred" OR "client-centered" OR "client centered" OR "decision-making" OR "decision making" OR "goal setting" | "occupational therapy" OR "occupational therapist" OR "occupational therapists" | pediatrics OR  child* OR pediatric OR paediatric OR youth* OR toddler* OR adolescent* OR infant | | qualitati* | - Subject area: Health Professions - Language: English - Document type: Article - Year: 2005-2022 - Publication stage: Final |

*Note.* Terms in bold are database-specific controlled vocabulary.
