# Supplementary material for: Exploring Therapeutic Relationships in Pediatric Occupational Therapy: A Meta-Ethnography
Source: Can J Occup Ther. 2023 Jul 4;91(1):78–87. doi: 10.1177/00084174231186078 (PMC10903134; doi:10.1177/00084174231186078)
Supplement: sj-docx-2-cjo-10.1177_00084174231186078 - Supplemental material for Exploring Therapeutic Relationships in Pediatric Occupational Therapy: A Meta-Ethnography [file sj-docx-2-cjo-10.1177_00084174231186078.docx]

**Table**

*Data Extraction Table for Studies Included in the Meta-Synthesis*

| Authors | Year | Location | Aim | Clinical settings | Participants | Design | Data collection and analysis | Main findings | Quality |
| --- | --- | --- | --- | --- | --- | --- | --- | --- | --- |
| King, Chiarello, Ideishi, Ziviani, Phoenix, McLarnon, Pinto, Thompson, & Smart | 2021 | Canada and United States | To understand the meaning of therapy interactions from the viewpoint of youth, caregivers, and service providers | Outpatient rehabilitation | Children (n = 13); caregivers (n = 15);  service providers (n = 26) | Ethnography | Data collection: Observations and interviews  Data analysis: Thematic analysis | The main themes were “expectations influenced engagement/disengagement and progress in therapy”, “engagement was associated with positive affect and relationships”, and “engagement was strongly associated with relationships and collaboration”. | KP |
| Keiter Humbert, Cargill, Sanders, & Wood | 2021 | United States | To understand how occupational therapists provide care for families in complex socioeconomic and cultural contexts | Early intervention, school and home-based services | Occupational therapists (n = 8) | Phenomenology | Data collection: Interviews  Data analysis: Thematic analysis | The main theme was “We're in This Together”. The subthemes were “It Takes Time”, “Navigating Therapeutic Conversations and Highlighting Successes”, “Going Above and Beyond”, “Include Everybody”, and “Learning From One Another”. | SAT |
| O’Connor, Lynch, & Boyle | 2021 | Ireland | To describe the decision-making experiences of children, parents and  therapists in occupational therapy | Public health-care system and private clinics | Children (n = 6); caregivers (n = 5); occupational therapists (n = 6) | Qualitative descriptive | Data collection: Interviews  Data analysis: Thematic analysis | The themes were “Goal-setting experiences” (“The who, how and what of goal-setting in pediatric occupational  Therapy”, and “Decision-making: Shared or restricted?”), “Adults: child-rights gatekeepers or defenders?” (“Knowledge, views, and attitudes”, and “Power and influence in pediatric occupational therapy”) and “Decision-making in context” (“The “fight” for services”, and “Aspirations and Actuality”). | KP |
| Kennedy, Missiuna, Pollock, Whalen, Dix, & Campbell | 2020 | Canada | To describe the factors that therapists view as influencing the development of family–therapist relationships in Partnering for Change school-based services | School-based services | Occupational therapists (n = 15) | Qualitative descriptive | Data collection: Focus groups  Data analysis: Content analysis | The categories were “Competing demands”, “Consistency and availability”, “Awareness, readiness  and commitment”, “Relationship with schools and educators”, and “Sociodemographic factors”. | SAT |
| Reeder & Morris | 2018 | United Kingdom | To understand the views and experiences of pediatric therapists working in the regarding the importance of the therapeutic relationship when providing information for parents of children with long-term disabilities | Public health-care system | Service providers (n = 7) | Phenomenology | Data collection: Interviews  Data analysis: Thematic analysis | The main theme was “The Importance of the Therapeutic Relationship: Balancing a Positive Relationship with Professional Responsibility”. | SAT |
| Smit, de Jongh, & Cook | 2018 | South Africa | To provide descriptions and vicarious experiences of what it was like for parents of a child with sensory integration disorders receiving occupational therapy | Unclear | Caregivers (n = 9) | Phenomenology | Data collection: Interviews  Data analysis: Creswell’s multiple level of analysis | The main theme was “Just suddenly everything made so much sense”. The subthemes and categories were “Role of the occupational therapist” (“I’m a simple act. I like to know why: Parent education and reframing”, and “The trust factor…it just speeded everything up”. Collaboration, accessibility and trust”) and “OT/SI intervention process (“No better lesson: Parent joining sessions”, “Emotional rollercoaster ride: progression and regression of OT/SI intervention”, “Jargon use”, “Practical strategies and home programmes”, and “Financial cost and length of intervention”. | FF |
| Kruijsen-Terpstra , Verschuren, Ketelaar, Riedijk, Willem Gorter, Jongmans,& Boeije | 2016 | Netherlands | To explore the experiences and needs of parents of young children with cerebral palsy regarding their child’s physical and occupational therapy process in a rehabilitation setting | Outpatient rehabilitation | Caregivers (n = 21) | Qualitative descriptive | Data collection: Interviews  Data analysis: Thematic analysis | The main themes were “Information” (“Cerebral Palsy in general”, “Therapy”, and “Prospects”), “Communication” (“Attentiveness”, and “Honesty”), and “Partnership between parents and therapists” (“Roles” and “Involvement”). | SAT |
| Shea & Jackson | 2015 | United States | To explore how at-risk youth respond to client-centred and occupation-based occupational therapy in the community | Community-based services | Children (n = 5) | Phenomenology | Data collection: Interviews  Data analysis: Interpretative phenomenology analysis | The main themes were “the client-centered and occupation-based OT intervention process”, “the youth’s increased self advocacy”, and “the youth’s enhanced perception of their future”. | FF |
| Tam-Seto & Versnel | 2015 | Canada | To understand shared decision making in adolescent mental health using the Canadian Model of Client-Centered Enablement | Mental health services | Occupational therapists (n = 6) | Qualitative study rooted in the critical incident technique | Data collection: Interviews  Data analysis: Thematic analysis (unclear) | The main themes were “Role of therapist: Supporter, Collaborator, Decision maker”, “Client factor: Mental health disorder, Role of family, Cognitive skills, Client readiness”, “Therapeutic relationship: Rapport, Transparency” and “Nature of decision: Risk level”. | SAT |
| Lindsay, Tétrault, Desmaris, King, & Piérart | 2014 | Canada | To explore how occupational therapists act as cultural brokers in working with immigrant families who are raising a child with a physical disability | Services from child developmental programs | Occupational therapists (n = 17) | Descriptive qualitative | Data collection: Interviews  Data analysis: Thematic analysis (unclear) | The themes were “Constraints” (“Cultural constraints” and “Socio-structural constraints”) and “Strategies to Overcome Constraints” (“Translating between health systems”, “Bridging different meanings of occupational therapy”, “Establishing long-term relationships”, and “Working with clients’ relational networks”). | KP |
| Andrews, Griffiths, Harrison, & Stagnitti | 2013 | Australia | To explore how occupational therapists' approach working with families from low socio-economic positions | Early intervention services | Occupational therapists (n = 9) | Qualitative descriptive | Data collection: Interviews  Data analysis: Thematic analysis (unclear). | The main themes were “We work with families in different ways” and “Parents' needs and expectations – from child focussed to family focused. | SAT |
| Fingerhut, Jocelyn Piro, Ashley Sutton, Rachel Campbell, Christy Lewis, Dilshad Lawji, & Nicole Martinez | 2013 | United States | To explore how family-centred care is implemented by occupational therapists providing intervention for children | Home based, clinic based, and school based | Occupational therapists (n = 28) | Grounded theory | Data collection: Interviews  Data analysis: Axial coding in grounded theory | The main categories were “Communication With Parents”, “Incorporating Elements of Family-Centered Practice”, “Barriers to Family-Centered Practice”, and “Family Outcomes”. | SAT |
| Kolehmainen, Duncan, McKee, & Francis | 2010 | United Kingdom | To explore caregivers’ views of the interactions with therapists | Unclear | Caregivers (n = 7) | Qualitative descriptive | Data collection: Interviews  Data analysis: Thematic analysis | The main themes were “Actively to ‘do things’ to support children”, “Issues of access”, and “Interactions between therapists and the parent, the child and educational staff”. | SAT |
| Nelson & Allison | 2007 | Australia | To investigate what constitutes a socially and culturally appropriate occupational therapy service for urban Indigenous children | Unclear | Occupational therapists (n = 50) | Qualitative descriptive | Data collection: Survey  Data analysis: Thematic analysis | The main themes were “Developing effective relationship” (“Have a consistent therapist”, “Use occupation as a context to help address communication”, “Relationships improve outcomes”, “Use relationships to engage the whole family”, “See children in groups”, and “Use existing relationships”), “Develop personal qualities required for practice”, and “Address logistical issues of service delivery”. | FF |

*Note.* Included study was evaluated as a key paper (KP) with conceptual richness and rigour in the method; a satisfactory paper (SAT); or a paper that is “fatally flawed” from a methodological standpoint (FF) as proposed by Malpass et al. (2009).
